# The 5As team patient study: patient perspectives on the role of primary care in obesity management

**DOI:** 10.1186/s12875-017-0596-2

**Published:** 2017-02-08

**Authors:** Jacqueline Torti, Thea Luig, Michelle Borowitz, Jeffrey A. Johnson, Arya M. Sharma, Denise L. Campbell-Scherer

**Affiliations:** 1 Department of Family Medicine, University of Alberta, Clinical Research Unit, Edmonton, AB Canada T6G 2E1; 2 School of Public Health, University of Alberta, Edmonton, AB Canada T6G 2E3; 3 Department of Medicine, Division of Endocrinology, University of Alberta, Edmonton, AB Canada T6G 2E1; 4 Department of Anthropology, University of Alberta, Edmonton, AB Canada T6G 2H4; 5 Department of Medicine, Obesity Research & Management, University of Alberta, Li Ka Shing Building, Rm. 1-116, 87th Avenue and 112th Street, Edmonton, AB T6G 2E1 Canada

**Keywords:** Obesity, Primary health care, Qualitative research, Family medicine

## Abstract

**Background:**

Over 60% of people have overweight or obesity, but only a third report receiving counselling from primary care providers. We explored patients’ perspectives on the role of primary care in obesity management and their experience with existing resources, with a view to develop an improved understanding of this perspective, and more effective management strategies.

**Methods:**

Qualitative study employing semi-structured interviews and thematic analysis, with a sample of 28 patients from a cohort of 255 patients living with obesity and receiving care to support their weight management in a large Primary Care Network of family practices in Alberta.

**Results:**

Four illustrative themes emerged: (1) the patient-physician relationship plays an important role in the adequacy of obesity management; (2) patients have clear expectations of substantive conversations with their primary care team; (3) complex conditions affect weight and patients require assistance tailored to individual obesity drivers; (4) current services provide support in important ways (accessibility, availability, accountability, affordability, consistency of messaging), but are not yet meeting patient needs for individual plans, advanced education, and follow-up opportunities.

**Conclusions:**

Patients have clear expectations that their primary care physician asks them about weight within a supportive therapeutic relationship. They see obesity as a complex phenomenon with multiple drivers. They want their healthcare providers to assess and address their root causes - not simplistic advice to “eat less, move more”. Patients felt that the current services were positive resources, but expressed needs for tailored weight management plans, and longer-term follow-up.

## Background

Obesity is a prevalent, complex, chronic, relapsing condition associated with medical, genetic, psychological, socio-economic, and cultural root causes and drivers of weight gain as well as multiple comorbidities that require coordinated, long-term, and interdisciplinary care [1–6]. Recognizing this escalating challenge, the Canadian Medical Association declared obesity a chronic disease in 2015 [7]. Both the Canadian and US Task Forces on Preventive Health Care recommend that primary care practitioners screen patients, counsel regarding weight loss, and refer to structured behavioural interventions aimed at weight loss [8–10]. However, only 37% of Canadian patients report that they have received any counselling regarding diet and 24% report receiving any counselling regarding exercise [11, 12]. Many physicians feel ill-equipped to assist patients in weight management, and lack training in the management of obesity [3–5, 13–16]. Obesity counselling and multicomponent interventions in primary care settings, that for example include diet, exercise, and counselling or behavioural therapy, improve obesity prevention and management (referred to herein as weight management) [13, 17, 18]. Patients want their primary care providers to address their weight concerns [5].

The 5As Team program of research aims to address this gap. The program has partnered with a Primary Care Network (PCN) in Alberta, Canada, and worked with frontline interdisciplinary healthcare providers to help them improve their clinical practice [19–22]. As a component program, we wanted to explore patient needs and experience with PCN weight management resources to inform the results of the provider study, and to begin the process of designing an intervention. PCNs in Alberta have the mandate to enhance family practices with access to interdisciplinary teams to improve chronic illness prevention and care using a patient-centred approach [23]. To achieve patient-centeredness it is vital to understand patients’ expectations and experiences of healthcare services. We conducted a study answering the questions 1) From the perspective of patients who seek primary care support for weight management, what role do clinicians play in their efforts? 2) How do PCN resources offer meaningful support and satisfy patients’ needs? The purpose of this study is to lay the foundation for co-creation with patients of an intervention for a planned randomized control trial.

This paper presents the results of this qualitative study. Unique is our focus on patients’ perceptions of the role of primary care providers and PCN programming in overall weight management. This foundational research is needed to develop effective and sustainable interventions.

## Methods

The 5As Team intervention study (5AsT) is a longitudinal randomized control trial with convergent mixed-methods evaluation that has been described in detail elsewhere [19]. The qualitative sub-study recruited 28 patients from the 5AsT longitudinal patient cohort. Patients were over 18 years old, had given written informed consent in English, and had a BMI > = 25 kg/m^2^. Purposeful sampling targeted individuals who received care of a PCN dietician or participated in a group program to support weight management at least 6 months prior. This ensured that patients were able to comment on the utility of PCN resources for their weight management. Over a 9-week period MB conducted qualitative interviews following a semi-structured interview guide (Appendix 1). The University of Alberta’s (U of A) Health Research Ethics Board (HREB) provided institutional ethics approval (Pro00036740).

Interviews were transcribed verbatim, edited for clarity and confidentiality, and formatted for analysis. We used thematic analysis and iteratively coded data revising our list of codes continuously based on emergent patterns that were discussed regularly in analysis discussions with the research team [24, 25]. To ensure validity we used analyst triangulation that included the development of a coding scheme between three investigators (JT, MB, DCS), as well as independent coding of each interview by two investigators (JT, MB). Codes were cross-checked and differences discussed to reach consensus. Emergent themes were critically discussed with the third author (TL) to maintain reflexivity and minimize bias [26]. To maintain confidentiality we used pre-established codes to replace formal names of interviewed patients, primary care providers, and family practice clinics.

## Results

A total of 28 individual interviews were conducted with patients from a variety of ages, sexes, socioeconomic status and medical co-morbidities (Table 1). While interviews illustrate the individual character of patients’ weight management journey, four themes emerged that reflect the commonalities in perspectives, expectations, and needs across all patients:

1. *Coordinated and person-centred care*: Patients emphasize the need for coordinated, whole-person approaches to address the multiple conditions and drivers that affect weight and weight management;
2. *The role of family physicians*: Patients have clear expectations of their primary healthcare clinician to initiate a discussion around weight concerns;
3. *Primary Care Network resources for weight management*: PCN programming and services meet patients’ expectations for weight management support by being accessible, providing accountability, and contributing to consistent weight management messaging;
4. *Patients’ weight management needs*: Patients express further needs for weight management supports that are individualized to their medical, socio-economic, and educational needs, better linkages to available resources and programs, and further follow-up.

**Table 1:** Participant characteristics and demographics

Age range (years)	*n* (%)
30–45	4 (14)
46–60	12 (43)
61–75	11 (39)
> 75	1 (4)
Median age = 58 (range 30–80 years-old)	
Sex	*n* (%)
Female	19 (68)
Male	9 (32)
Ethnicity	*n* (%)
Caucasian	27 (96)
Aboriginal	1 (4)
Chronic Disease	*n* (%)
Arthritis	12 (43)
Hypertension	12 (43)
Hyperlipidemia	11 (40)
Asthma	7 (25)
Diabetes	7 (25)
COPD^a^	2 (7)
Heart Disease	2 (7)
Kidney Disease	1 (4)
Income	*n* (%)
$15,000–$29,000	5 (23)
$30,000–$49,000	4 (18)
$50,000–$79,000	8 (36)
> $80,000	5 (23)

^a^(Chronic Obstructive Pulmonary Disease) COPD

### Coordinated and person-centred care

Patients described their weight management efforts as inseparably tied to other physical, mental, and socio-economic conditions. These conditions pose significant barriers to patients’ weight management efforts causing feelings of frustration with their ability to reduce weight (Table 2). Patients emphasized that they needed their providers to adopt a comprehensive perspective addressing their obesity in the context of patients’ comorbidities and life circumstances that affect health and their ability to manage weight:

*“So, I try to work on one aspect of my life. The stressors in my life at the time. But I need something to pull it all together. Yeah. It would seem reasonable that I should be able to find help within the healthcare systems.…somebody with a professional background to say, ‘Okay, you need help in this area. You need to put all this together now’.” (Patients 27)*

**Table 2:** Conditions and drivers affecting weight and weight management

Subtheme	Patient Quotes
Mechanical	• I blew my knee out for the first time probably in the late nineties and that’s when my weight really started to go up. ‘Cause that reduced my physical activity I was doing. (Patient 3)
	• This cough…I just feel that I’m not getting air into my lungs. No ending it. I thought it was a cold, but then after a couple of bottles of cough syrup and no relief, he (family physician) said, ‘Throw it away, here’s an inhaler.’ I’ve done lung capacity and all kinds of x-rays, and done the sinus and the whole thing and nothing. . .Yeah, snowballs. I think there’s a bit of a steroid in this inhaler, because I’ve noticed the weight has gone up since I started on the inhaler. (Patient 6)
Mental	• I was seeing my physician and we were talking about me being an emotional eater… (Patient 4)
	• I have other issues that relate to the weight because I’m an alcoholic, we’re dealing with all of that together. So that’s a big part of my weight management is quit (chuckle) drinking. So she’s more concerned about that issue than she is about my weight. Because if I stop drinking, the weight is going to come off and I’m noticing that. (Patient 16)
	• . . .because of the weight gain that I’m having. And the stress that’s in my life! (chuckle) The two go hand in hand. (Patient 27)
	• My weight issues are connected to mental and emotional issues from childhood so there is a mental component, sort of almost borderline addiction component to my obesity… (Patient 22)
Metabolic	• I can tell you where I got pretty heavy at one point, really heavy, and that’s due to the fact I went through prostate cancer. Also, I’ve taken radiation, and then hormone therapy to knock down your testosterone, which makes you gain weight. . . They just told me it was a common thing to take place when you’re taking hormones. So, don’t panic! (Patient 10)
Monetary	• When I get paid I go grocery shopping. But I get to a point where I don’t have any money. So, I might have to call the Food Bank. Well, last month, I unfortunately had to call the Food Bank. What I got in my basket was bagels, three loaves of bread, cereal, one lettuce, tube things of pasta, two bags of carrots, and a bunch of sweets. Nothing that I could eat except the lettuce! So when you’re hungry it’s hard because that’s all you have. I hardly ever buy canned food, because there’s too much garbage in it. If I do I make sure it’s things, like tuna, that I know I can eat. I try and keep things like that for my emergencies. But it’s really hard when you have no money and you need food. What do you eat? (Patient 16)

Patients explained how painful conditions, such as knee injuries, chronic pain, asthma, osteoarthritis, and plantar fasciitis, impaired their mobility and reduced the amount and level of their physical activity. Some patients reported weight gain from prescription medication, and other mechanical concerns such as sleep apnea. Patients also shared personal stories of their challenges and struggles with obesity and mental health. In these stories, patients link their weight, eating and weight control behaviours to stressful life events, depression, traumatic childhood experiences, fatigue, stress, and addictions. In addition, patients identified Type 2 diabetes, pre-diabetes, hypertension, and gout as associated with their weight. Women described struggling with the effects of menopause. These metabolic conditions often required restricted diets that patients found difficult to realize in their everyday lives. Finally, patients discussed limited finances and time constraints that affect their means to maintain or commit to an obesity management program.

Patients are aware of the connection between their overall health, socio-economic well-being, and comorbidities with their overweight, and they look for support to assess, prioritize, and plan their weight management holistically.

### The role of family physicians

Patients had clear and consistent expectations concerning the role of primary care in their weight management. They believed that it is the responsibility of the family physician to initiate the conversation about weight management, and to do so in a non-judgmental, knowledgeable and respectful manner. They felt that the conversation should be guided by a holistic view of weight that acknowledges the patients’ overall health and well-being.

Patients discussed how aspects of their encounter with family physicians affect their satisfaction with weight management support (Table 3). These included perceived quality of relationship with the physician, the physician’s approach to weight management and knowledge of obesity, and the time spent on the topic.

**Table 3:** The role of family physicians

Subtheme	Patient Quotes
Physicians should initiate weight management conversation	• “I think that having that conversation would be beneficial. I mean even though things are going well now, in the future this could be potentially what being overweight might cause. I think that would be a huge help for a lot of people. It would be a help for me too.” (Patient 14)
Physicians should not be judgemental	• “I think a concerned family physician should always talk to you about your weight management, but not denigrate or harass you about it. Because you’ve achieved this for some reason…You won’t change it by medicine, you’ll change it by attitude and life.” (Patient 15)
Positive experience	• “I love my new doctor because he actually says stuff to you, very nicely. He’s very kind and gentle about it but he’ll actually say, ‘It’ll help if you lose weight.’ And he encouraged me to come here and encouraged me to take more classes.” (Patient 21)
	• “My doctor doesn’t deal with the weight management as a completely separate issue. It’s affecting my health, my recovery and my ability to exercise. She sees me once a month for a follow-up appointment to check my prescriptions, how I’m feeling, and my weight. So it’s part of the bigger picture.” (Patient 7)
Negative experience	• “My family physician has never ever talked about my weight.” (Patient 5)
	• “My family physician would give me the standard, ‘Eat less fat, eat less sugar (chuckle), exercise more,’- and that’s not going to cut it” (Patient 2)
	• “They were like, ‘Okay, cut your calories and exercise,’ and that was their only thing… Eat less and exercise more isn’t the end-all-be-all. I’m looking for more concrete strategies.” (Patient 11)

Some patients reported a positive doctor-patient relationship with their family physician, were at ease discussing their weight, and believed their physician had listened and responded to their needs and concerns. A number of patients perceived their physician as effectively communicating weight management options. In addition, patients appreciated physicians who considered their overall health rather than addressing weight in isolation of other health concerns.

Other patients stated that their family physician never discussed weight. Some patients attributed this to a lack of time during the consultation. Others felt their family physician either ignored or did not recognize their weight concerns and the effect it is has on not only their health and wellbeing, but also their family, social, and work life. Some patients felt that their physician’s failure to address weight was due to the patient’s general good health. These patients, however, would have preferred their physicians’ respond to their concerns and willingness to discuss weight management as a preventative measure. Several patients reported that their physicians expressed weight management was outside their field of expertise and referred them to a dietician or chronic disease management nurse. Some patients experienced their physician’s approach to weight management as a variation of ‘*eat less and move more*’, which they found unhelpful and ineffective.

### Primary care network resources for weight management

Patients described participation in the PCN educational sessions (Table 4) as useful and beneficial for weight management. Several patients had one-on-one support from dieticians in the PCN. Patients explained that these PCN resources facilitated their weight management through three key elements: *accountability, accessibility, and consistency* (Table 5).

**Table 4:** Examples of some PCN educational programs

Program Title	Duration	Purpose
Weight Management	Full Day or 3 weeks	Information on safe dieting techniques, emotional eating, and setting achievable weight loss goals
Moving for Health	8 weeks	Geared towards people living with chronic disease who want to incorporate exercise into their life to improve health
Grocery Shopping	1 day	Coaching in making better food choices: planning meals, reading nutrition labels, and grocery-shopping tips
Meal Planning	1 day	Coaching in making better food choices: planning meals, reading nutrition labels, choosing healthy take-out options.
Relaxation	4 weeks	Strategies to help patients cope with everyday stress and stress related to various health problems
Managing Emotion	Full Day or 3–4 weeks	Helps patients recognize their strengths and weaknesses to effectively manage stress and choose healthy behaviours
Changeways	6 weeks	Strategies for managing depression and anxiety

**Table 5:** PCN programming meets patients’ expectations in important ways

Subtheme	Patient quotes
Accountability	• Well, I think it (regular attendance of PCN programs) sort of keeps you accountable a little bit when it’s a regular thing, you know. (Patient 8) • Like certain things like how many laps you could do in a circle… And they would compare it when you’re done the workshop at the end of the eight weeks, I think…. And I noticed I did increase because I think it held me accountable. (Patient 1)
Accessibility	• I’m glad that they did evening sessions because then I could go. (Patient 16)
	• They seemed to really let you know what they had available, that they wanted to be able to help you out, which is nice. (Patient 14)
	• Well I’m just totally happy with PCN. I just think it’s like a gold mine! Like, you know. And it’s really nice to have…like services are, you know, not real expensive or free here. (Patient 2)
	• Maybe I’m just more appreciative that it doesn’t cost me any money, so psychologically I have been more acceptable to the (PCN) program. I feel it’s a caring kind of a thing, not a business kind of a thing. (Patient 11)
Consistency	• I felt that they were all pulling together. They weren’t necessarily communicating deeply with one another, but I never felt like I was getting conflicting messages. The message was always the same. What one person was saying was building…I could take what the doctor said, what the dietician said, what the PCN people said, and what the exercise people said and put it all together and build on it. There was no confusion. (Patient 27)

#### Accountability

Patients expressed that their participation in the programs and the interactions with staff helped to hold them accountable when it came to weight management. They emphasized accountability as a major benefit of using PCN resources. For example, some patients stated regularly scheduled sessions alone increased their commitment. Other key benefits of these programs included explicit goal setting and deliberate follow-up with a PCN clinician to discuss successes and challenges that the patient experienced. Patients expressed that setting goals and working towards achieving them as best as they could, helped them to maintain their motivation.

#### Accessibility

A major strength of the PCN supports was their accessibility including availability of interdisciplinary clinicians, accommodating schedules, and affordable cost. Patients described benefitting from access to nutritionists, exercise specialists, and behavioural health consultants that focused on underlying psychological issues and behavioural change. In addition, they benefited from access to clinic nurses, which assisted with healthy weight loss and weight maintenance. However, patients also expressed that despite dietician availability, access is insufficient because of lengthy waiting times and lack of capacity to provide individualized support and follow-up.

Weight management sessions were offered after work and on weekends, with PCN clinicians available to patients beyond regular scheduled sessions. In addition, an Alberta Health grant funds the PCNs, as such program fees are either *gratis* or nominal, which considerably lowers the financial barriers to participation. Having affordable access to these weight management resources, patients perceived the PCN as more “caring” and patient-centered as compared with commercial weight loss programs. Patients’ experience of the PCN as caring played a significant role in their decision to access the PCN as a weight management resource.

#### Consistency

Another element of PCN resources and supports that emerged as significant for patient satisfaction was consistent weight management messaging. Patients remarked that a core point in this messaging was that weight loss is not a ‘quick fix’ and they should consider their weight management as a journey that will take commitment and time.

### Patients’ weight management needs

Four additional areas of weight management support were identified: (1) more individualized weight management resources; (2) addition of more advanced educational sessions; (3) enhanced advertisement; and, (4) further follow-up (see Table 6).

**Table 6:** Further patient needs for weight management

Subtheme	Patient quotes
Individualized weight management resources	• …when you do a group session, there are people with so many different dietary needs. There were a lot of people in that class that had…were looking at needing bariatric surgery and all kinds of other things. So the needs of all people were so different that it’s hard to get the answers to what you want because everybody is different. (Patient 16)
Advanced educational sessions	• I would like there to be more of the *Moving for Health*. It would be nice if there was different levels, because they’ve got a lot of equipment there. It would be great to learn how to use. There’s just that first one to get you moving and then it’s up to you to carry on. It would be great if they would do classes. I think a lot of people would come here to do it because one if it’s free, and two when everybody else is overweight, you’re more comfortable. You’re not as comfortable when you’re having to go to a gym and everybody is all healthy and skinny and running on that track, so if you could come here and do Jazzercise or that kind of thing in the gym. (Patient 19)
Enhanced advertisement	• I think the one thing that I would say was if this were more readily advertised…The PCN programs were advertised that they were available, I think I would have jumped on them a lot faster ‘cause it is something that is interesting and motivating for me. (Patient 14)
Follow-up sessions	• The other thing that would be really helpful in these programs is the commitments from patients. Again, it comes down to accountability. So the patient says, ‘I’m going to do this, this, and this.’ When you check back, whoever it is that you’re checking in with is asking, ‘Have you done this?’ ‘Why?’ ‘Why not?’ ‘How did it work?’ ‘What can you do further?’ ‘If that’s not work, what can we do instead?’ (Patient 23)
	• The other part would be accountability. That there is an opportunity either at the PCN or at the doctor, whoever wants to take the lead that can be an ongoing follow-up. I think the programs are great, but when the program ends, what happens next to that patient? . . . And, the thing is this is a lifestyle thing. So all of those things are great building blocks on your lifestyle, but what is going to keep you working on that lifestyle, to improve in general. So, it would be an ongoing connection, I think, with a PCN or with the doctor. I think PCN would be a great place to continue because they have some resources that the doctor’s offices don’t always have. (Patient 23)
	• I’d love something that could be ongoing! Almost like a course where it was a whole year long and you make a commitment to it…Yeah I’d love something like that where you’re more accountable, there’s more follow up, and you can ask questions and express your concerns. (Patient 2)

### Strategies for individual sets of comorbidities

Patients had a good understanding of the multifactorial issues at play in their weight management and expressed the need for resources tailored to their particular situation. While patients appreciated that group programs provided a venue to share experiences with other patients, they found that the content remained too broad, thus not providing strategies that are applicable to their particular situation. They suggested that increasing the opportunity for one-on-one interaction would help them to address their individual weight management challenges.

### Advanced weight management knowledge

Although patients understood the broad nature of the educational sessions and appreciated that they start at a very basic level, they felt that they would also benefit from more advanced educational resources.

### Linking and advertising resources

Patients indicated the need for better promotion and advertisement of weight management resources and programs. Improved descriptions of programs would allow patients to make informed decisions about which resources would benefit their weight management best.

### Follow-up

Patients expressed that extended follow-up with primary healthcare providers would improve their overall weight management experience, increase patient and provider accountability to meet their weight management goals, and help address any challenges patients may be experiencing. In addition, sometimes patients felt overwhelmed with the volume of information given in one consultation and would prefer long-term weight management resources. They felt ongoing access to resources and programming would be supportive in establishing new and improved eating behaviours, and encourage commitment to sustainable behaviour change.

## Discussion

Family physicians should play a vital role in weight management, which is consistent with current literature [3, 13, 15, 17, 27, 28]. Physicians should play a dual role in initiating weight management consultations with a comprehensive assessment and coordinating care of conditions and comorbidities that affect weight. In addition, we found that PCN services support weight management in important ways that meet patient needs for accessibility, accountability, and consistency. Finally, the results point to where patients’ needs are not met and future efforts are warranted.

Patients clearly articulated that they would like their family physician to initiate weight counselling, which is consistent with existing literature [3, 5, 16, 29]. However, our findings emphasize the need to assess root causes and drivers of weight gain, comorbidities, and psychosocial barriers of individual patients in order to develop a care plan that offers realistic and sustainable strategies according to patients’ particular constraints and strengths. Patients felt that their weight is a complex phenomenon impacted by a multitude of factors. Consistent with literature that categorizes common conditions associated with obesity into the “4Ms of obesity management” [6], patients discussed mechanical, mental, metabolic, monetary issues as inseparable from their weight trajectory and has having decisive impact on their weight management efforts. This resonates with the mounting evidence showing that obesity is a complex condition that is impacted by multiple factors beyond diet and exercise [3, 5, 16, 30, 31]. Consequently, weight management in primary care must build on an individual assessment of a person’s history with weight, attempts at weight control, comorbidities, mental health, and socio-economic situation. Findings underscore that patients want personalized information about physiology of obesity, its chronicity and resistance to treatment in order to formulate personal strategies with realistic expectations, and a focus on prevention and patient-important health outcomes rather than numbers on a scale. In addition, treatment plans must be guided by the recognition that behaviour and behaviour change arises at the intersection of cultural and social environments with individual factors such as automatic responses, conscious choice, values, and physiology [3, 5, 32, 33]. Family physicians are ideally positioned to develop such personalized care plans based on their long-term relationship with patients and the lifecycle approach of primary care.

Our findings show that while patients expect physicians to initiate and coordinate weight management; extended interdisciplinary health teams contribute valuable care and resources beyond the constraints of physicians’ time and expertise. This echoes previous research calling for multidisciplinary teams to address obesity [3, 5, 15, 16].

Patients appreciated the sense of accountability to their weight management goals through PCN programming. The importance of accountability for behaviour change has been found previously and points to the relational quality of human behaviour [2, 32]. Goal setting and follow-up, in concert with social support, facilitates the establishment of healthy habits [34, 35]. Patients value consistent messaging around prioritizing health improvement instead of focusing on weight alone, realistic expectations for weight loss, and the holistic understanding of obesity. Such consistency in conceptualizing and framing weight management for patients supports their sense-making of their condition and their ability to manage it [21]. These findings support the potential of the Patient Medical Home model, that the PCN structure is based on for improving weight management and obesity care [23, 36].

Beyond these benefits of current weight management resources, patients emphasized the lack of personalized care plans including information that applies to patients’ particular combination of challenges and offers knowledge that is appropriate for diverse levels of education. They also emphasized the need for long-term, sustainable approaches and for better coordination of existing resources in the community. Challenges to address these needs include a lack of physician education in obesity physiology and training in weight management skills resource constraints for patient-centeredness of the current health care model and the lack of an evidence-based, yet flexible and adaptable, intervention [3, 13, 17, 29, 30].

Practice implications of our findings include a clear need for personalized weight management interventions that are practical and sustainable for interdisciplinary primary care clinicians. The “5As for obesity management™” (Ask-Assess-Advise-Agree-Assist) offer a promising framework that can guide practitioners in addressing patients’ individual weight management needs in a way that is patient-centred, comprehensive, and coordinated [13, 15, 21, 22, 27, 37]. The 5As are gaining traction in the US and Canada [13, 15, 19, 27, 37–40], have been shown to improve provider self-efficacy, increase weight management visits [13], and result in clinically significant patient weight loss at 12 months post-intervention with a physician trained in the 5As [24]. However, considering the chronicity and complexity of obesity as well as research questioning the utility of weight loss as measure for successful management [41], there is a pressing need for further research identifying appropriate patient outcome measures.

Furthermore, research is needed to understand how a comprehensive, personalized assessment and weight management plan can be implemented in diverse health care settings, in particular where extended interdisciplinary teams are not available and budget is limited, and how to build capacity in other interdisciplinary team members to support patient weight management.

There are some limitations of this study. All patients in this cohort had a family physician and received PCN weight management care. While 85% of Canadians have a family physician, and over 80% of Albertans belong to a PCN, the services and structures vary across different geographic regions. Although we achieved diversity of patients for gender, age, and stages of obesity, the majority of the group identified as Euro-Canadian and as urban/suburban residing in a major city in Alberta. The themes that emerged from the data reflect the perspectives of patients who are concerned about their weight and willing to work with clinicians to improve their health. While our conclusions are not generalizable to all patients living with obesity, they are foundational to developing an intervention that is effective for patients who seek support for their weight management within primary care.

## Conclusion

These results further our understanding of patients’ perception of if, when, and how primary care clinicians and resources can support weight management. The study is part of the larger 5AsT research program to improve weight management in primary care. Moving beyond patient experiences of living with and managing excess weight, this research examined how patients perceive the weight management support provided by extended interdisciplinary teams embedded in family medicine practices, such as the PCN model in Alberta, Canada.

Overall, patients expressed the need for weight management that is tailored to their individual set of comorbidities and provides strategies that are realistic and sustainable under their personal circumstances.

## Appendix 1: Interview guide

To provide some *background*, I would like to hear about *your weight management experience*. So, can we begin with…

1. Where do you go or where have you gone to get help with weight management?
  - What professionals, programs or resources have you used for weight management?
  - What types of healthcare providers, if any, have you talked to for weight management?
    i. Prompt - If not mentioned: Have you talked to your family physician or their clinic team about weight management?
    ii. Prompt - If not mentioned: Have you talked to PCN staff (nurse, dietician, mental health worker, behavioural health consultant, exercise specialist) about weight management?

### Family practice office

2) Tell me about your experience with your family physician including with members of their clinic team when you’ve talked about weight management.
  - What types of programs or resources were you provided with OR referred to for weight management?
  - What healthcare providers have you seen at your family physician’s office for weight management?
  - What types of programs or resources would you like from your family physician and/or clinic-based team to support your weight management? If no programs or resources, why not?
  - Were/Are you comfortable talking about your weight and weight management with your family physician and/or their clinic team?
  - Do you think your family physician and/or clinic team should talk with you about weight management? If yes, when should this occur? If not, why?

### PCN healthcare providers, weight management programs and resources

3) I would like to hear about your experiences with the PCN healthcare providers and their programs and resources for weight management.
  - How did you come to use PCN programs and/or resources for weight management?
  - What type of PCN programs have you used to support your weight management?
  - Are you using other PCN programs and/or resources for your health?
    i) If yes, are these a part of your weight management?
  - Are there PCN programs and/or resources that you would like to use to support your weight management but do not?
  - Are/were you comfortable talking about your weight with PCN healthcare providers?
  - What PCN healthcare providers have you talked to about your weight management?
    i) How are they providing the help you want in your weight management?
  - Are there PCN healthcare providers who you have not had access to but would like to talk to about your weight management?
    i) How would you want them to help you in your weight management?

### Additional questions

4) Thinking to a time before you talked about weight management with your family physician and their clinic team and/or PCN healthcare providers would you have considered primary healthcare providers as a resource to help you with weight management?
5) Do your family physician and PCN staff work together for your weight management?
6) How similar was the information you were told by your family physician and PCN staff about managing weight and setting goals?
7) Do you receive information about weight management from specialists? If yes, is it similar information that you received from your family physician and/or PCN healthcare providers?
8) Ideally, thinking about your weight management in the context of your health, how would you like to see your family physician and/or PCN healthcare providers helping you with your weight management?
9) Is there anything you would like to tell me about your weight management experience that we have not discussed?

## Abbreviations

4M’s: Mechanical, Mental, Metabolic, Monetary issues
5A’s: 5A’s for Obesity Management (Ask – Assess – Advise – Agree – Assist)
5AsT: 5As team
BMI: Body mass index
HREB: Health research ethics board
PCN: Primary care network
UofA: University of Alberta
