# Erratum to: The 5As team patient study: patient perspectives on the role of primary care in obesity management

**DOI:** 10.1186/s12875-017-0631-3

**Published:** 2017-05-04

**Authors:** Jacqueline Torti, Thea Luig, Michelle Borowitz, Jeffrey A. Johnson, Arya M. Sharma, Denise L. Campbell-Scherer

**Affiliations:** 1 Department of Family Medicine, University of Alberta, Edmonton, AB T6G 2T4 Canada; 2 School of Public Health, University of Alberta, Edmonton, AB T6G 2E3 Canada; 3 Department of Medicine, Division of Endocrinology, University of Alberta, Edmonton, AB T6G 2E1 Canada; 4 Department of Anthropology, University of Alberta, Edmonton, AB T6G 2H4 Canada; 5 Alberta Diabetes Institute, University of Alberta, Edmonton, AB T6G 2T9 Canada

## Erratum

After publication of the original article [1], it came to the authors’ attention that the affiliations were incorrect.

Affiliation 1 (Department of Family Medicine, University of Alberta) incorrectly contained ‘Clinical Research Unit’, and the postcode was incorrect. Affiliation 5 (Alberta Diabetes Institute, University of Alberta) was mistakenly included as ‘Department of Medicine, Obesity Research & Management, University of Alberta’.

In addition, in the list of authors, Jeffrey A. Johnson should be affiliated to institutions 2 and 5, and Arya. A. Sharma should have been affiliation to institutions 3 and 5.

The list of authors and affiliations is correct in this erratum. The ‘Author details’ section of the article should have read as follows:

**Author details**

^1^Department of Family Medicine, University of Alberta, Edmonton, AB, Canada T6G 2T4. ^2^School of Public Health, University of Alberta, Edmonton, AB, Canada T6G 2E3. ^3^Department of Medicine, University of Alberta, Edmonton, AB, Canada T6G 2E1. ^4^Department of Anthropology, University of Alberta, Edmonton, AB, CanadaT6G 2H4. ^5^Alberta Diabetes Institute, University of Alberta, Edmonton, AB, Canada T6G 2T9.
